# Fully automatic characterization and data collection from crystals of biological macromolecules

**DOI:** 10.1107/S1399004715011918

**Published:** 2015-07-31

**Authors:** Olof Svensson, Stéphanie Malbet-Monaco, Alexander Popov, Didier Nurizzo, Matthew W. Bowler

**Affiliations:** a European Synchrotron Radiation Facility, 71 Avenue des Martyrs, CS 40220, 38043 Grenoble, France; b European Molecular Biology Laboratory, Grenoble Outstation, 71 Avenue des Martyrs, CS 90181, 38042 Grenoble, France; c Unit for Virus–Host Cell Interactions, Université Grenoble Alpes–EMBL–CNRS, Grenoble Outstation, 71 Avenue des Martyrs, CS 90181, 38042 Grenoble, France

**Keywords:** X-ray crystal centring, synchrotron instrumentation, macromolecular crystallography, automation

## Abstract

Considerable effort is dedicated to evaluating macromolecular crystals at synchrotron sources, even for well established and robust systems. Much of this work is repetitive, and the time spent could be better invested in the interpretation of the results. In order to decrease the need for manual intervention in the most repetitive steps of structural biology projects, initial screening and data collection, a fully automatic system has been developed to mount, locate, centre to the optimal diffraction volume, characterize and, if possible, collect data from multiple cryocooled crystals. Using the capabilities of pixel-array detectors, the system is as fast as a human operator, taking an average of 6 min per sample depending on the sample size and the level of characterization required. Using a fast X-ray-based routine, samples are located and centred systematically at the position of highest diffraction signal and important parameters for sample characterization, such as flux, beam size and crystal volume, are automatically taken into account, ensuring the calculation of optimal data-collection strategies. The system is now in operation at the new ESRF beamline MASSIF-1 and has been used by both industrial and academic users for many different sample types, including crystals of less than 20 µm in the smallest dimension. To date, over 8000 samples have been evaluated on MASSIF-1 without any human intervention.

## Abstract 2

A fully automatic system has been developed that performs X-ray centring and characterization of, and data collection from, large numbers of cryocooled crystals without human intervention.

## 1. Introduction   

Automation has been a key development in structural biology, from protein production and purification to crystallization, data collection and reduction, structure solution and model building (Aishima *et al.*, 2010 ▸; Arzt *et al.*, 2005 ▸; Beteva *et al.*, 2006 ▸; Bowler *et al.*, 2010 ▸; Dauter, 1999 ▸; Elsliger *et al.*, 2010 ▸; Ferrer *et al.*, 2013 ▸; Heinemann *et al.*, 2003 ▸; Holton & Alber, 2004 ▸; Kabsch, 2010 ▸; Monaco *et al.*, 2013 ▸; Ohana *et al.*, 2004 ▸; Panjikar *et al.*, 2005 ▸; Soltis *et al.*, 2008 ▸; Perrakis *et al.*, 1999 ▸; van den Bedem *et al.*, 2011 ▸). In combination with advances in molecular biology and computation, this has allowed not only large numbers of structures to be solved (Berman *et al.*, 2013 ▸) but also some incredibly challenging complexes to be revealed in detail (Ben-Shem *et al.*, 2003 ▸, 2010 ▸; Selmer *et al.*, 2006 ▸; Warne *et al.*, 2008 ▸; Zouni *et al.*, 2001 ▸). Efforts are now being aimed at the areas that still require manual intervention, in particular targeting the mounting of crystals on sample supports and the centring of crystals in an X-ray beam, two steps which still require considerable human involvement (Cipriani *et al.*, 2012 ▸; Deller & Rupp, 2014 ▸).

Fully automatic data collection at synchrotron sources has been discussed for many years within the macromolecular crystallography (MX) community, and efforts in automating the various steps have led to great advances over the last ten years. Developments in crystal-characterization software (Incardona *et al.*, 2009 ▸; Sauter *et al.*, 2004 ▸) and automatic sample changers (Cipriani *et al.*, 2006 ▸; Cohen *et al.*, 2002 ▸; Jacquamet *et al.*, 2009 ▸; Pohl *et al.*, 2004 ▸; Ueno *et al.*, 2004 ▸), in combination with standardization of sample mounts (Cipriani *et al.*, 2006 ▸), has greatly increased productivity. In addition to these developments, mail-in crystallography has been introduced at several synchrotron sites, with the pharmaceutical industry making the most use of these services (Malbet-Monaco *et al.*, 2013 ▸; Okazaki *et al.*, 2008 ▸; Robinson *et al.*, 2006 ▸). Pioneering efforts in the full automation of the data-collection process have been made at the LRL-CAT beamline at the APS, USA and the SSRL, California, USA, which offer an automatic service primarily for the pharmaceutical industry, but with a significant proportion dedicated to academic users (Wasserman *et al.*, 2012 ▸; Tsai *et al.*, 2013 ▸). However, as sample centring is based on optical image processing, tight restrictions on sample mounting are required in order to provide this service.

The increasing number of challenging projects and the movement of the academic community into the development of small-molecule inhibitor projects (Heikkila *et al.*, 2009 ▸) has led to a huge increase in the number of samples for MX, as screening both for crystal quality and for the presence of ligands has become more important. Many of the steps in this process are repetitive and, with modern software and detectors, can often be performed better automatically, especially in cases where crystals are embedded in opaque media and cannot be centred visually. This stimulated the development of a system with sufficient flexibility for fully automatic characterization and data collection from a wide variety of samples, allowing very few restrictions to be put in place. At the heart of this new system is a routine to locate crystals and centre optimal volumes to the beam. Automated routines based on scanning across the face of sample supports and coupling the output to data analysis (Aishima *et al.*, 2010 ▸; Bowler *et al.*, 2010 ▸; Cherezov *et al.*, 2009 ▸; Hilgart *et al.*, 2011 ▸) have been implemented at many synchrotron sources. This approach, variously known as mesh, grid or raster scanning, is highly effective in the location of crystals and in defining the best part of a crystal in one direction. However, these routines lack the ability to define the best diffraction volumes needed to be centred to the X-ray beam, and still require user input to define the mesh. Here, we describe routines to define a mesh area, locate the optimal diffraction volume within a support, or the crystals themselves, characterize the volume and subsequently collect an optimized data set according to user requirements. The routines are deployed on the new highly automated ESRF beamline MASSIF-1 and are reliant on well established technology to deliver a stable beam and new developments in sample handling (the RoboDiff sample changer and goniometer) developed at the ESRF (Nurizzo *et al.*, in preparation). In combination with the ESRF MX beamline environment, which includes automatic beam delivery (Gabadinho *et al.*, 2010 ▸), data processing and analysis (Brockhauser *et al.*, 2012 ▸; Incardona *et al.*, 2009 ▸; Monaco *et al.*, 2013 ▸), and a database (ISPyB) for sample tracking and data display (Delagenière *et al.*, 2011 ▸), the prospect of analysing many more samples than can be performed manually becomes possible, as the system runs unsupervised and presents data in an intuitive manner. With over 8000 user samples now processed, the system has been thoroughly tested and presents additional data from the consistent collection of information from multiple crystals from the same project. This allows the comparison of many parameters that are not usually recorded during experiments, such as exact crystal dimensions and diffraction variation within crystals (Bowler & Bowler, 2014 ▸).

The system is not designed to replace all user visits to the synchrotron, but rather to perform the repetitive work of screening crystals or collecting data, freeing researchers to spend time on more challenging data-collection problems or to study the underlying biology.

## 2. Experimental details and results   

**Figure 1:**
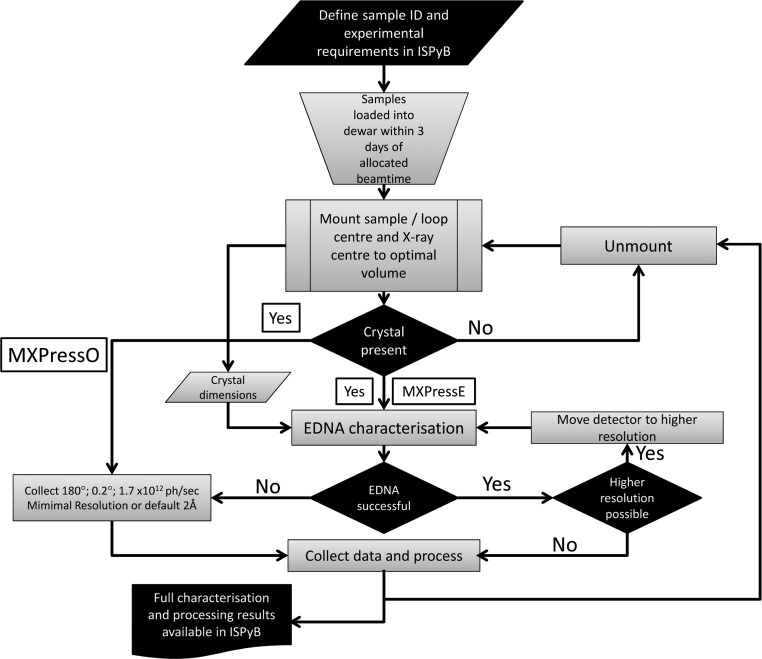
Graphical overview of the automated characterization and data-collection process running on MASSIF-1. All steps in the process are shown. The beamline-control GUI *MXCuBE* takes care of sample mounting/unmounting and optical centring of the sample mount. Information about the samples, dewar tracking and the presentation of results are *via* the beamline LIMS system ISPyB. The software described here starts once a sample has been mounted and optically centred to the X-ray beam.

The system presented here is based on earlier developments made in the automation of complex experiments (Brock­hauser *et al.*, 2012 ▸). The process is executed by a Passerelle workflow engine running on a server called the Beamline Expert System (BES). The BES is a customized version of Passerelle EDM (http://isencia.be/passerelle-edm-en) running on a central computing cluster independent of the beamline. The beamline-control GUI *MXCuBE*2 (Gabadinho *et al.*, 2010 ▸) starts the process by connecting to the BES *via* a web-service call. The workflow requests operations, such as data collections and motor movements, by connecting back to *MXCuBE*2 through an XML-RPC server. The process begins once a sample has been mounted and the loop optically centred; the steps and major decision points are shown schematically in Fig. 1 ▸. Each of the major steps is described in detail below.

### 2.1. MASSIF-1   

The automatic routine described here, and the modules such as X-ray centring, have been designed to run on any of the ESRF MX beamlines. However, it is only run as a dedicated service on the new highly automated beamline MASSIF-1. MASSIF-1 is a ESRF undulator beamline dedicated to the fully automatic characterization of and data collection from crystals of macromolecules. The beamline uses an artificial asymmetric Laue [110] diamond as a monochromator and a compound refractive lens (CRL) as the only focusing element. This simple optical setup produces a highly stable beam of 100 × 65 µm (H × V, FWHM) with a typical flux of 2 × 10^12^ photons s^−1^ at a fixed energy of 12.8 keV, allowing structure solution by single-wavelength anomalous diffraction (SAD). The beamline is equipped with a sample changer that also acts as a diffractometer (RoboDiff), a high-capacity dewar able to store 240 SPINE standard samples and a PILATUS3 2M detector. The beam diameter can be tailored to the crystal size using a series of apertures. The sphere of confusion (SOC) of the RoboDiff is less than 2 µm in diameter over 360°, allowing a minimum aperture of 10 µm to be used. The beamline will be fully described elsewhere (Bowler *et al.*, in preparation).

### 2.2. Automatic determination of mesh parameters   

Crystals vary widely in their morphology and can be mounted in a wide variety of supports. Optical centring will place the support at the beam position, but the crystal will not necessarily be in the beam over the full rotation range. It is therefore necessary to scan the loop through the X-ray beam in order to accurately locate small crystals and to define the optimum part of larger crystals. Initially, a fixed grid size was used. The fixed grid was sufficient to cover the largest loops available and also take into account that the loop might not be optimally centred. However, the fixed grid was substituted with an automatically determined grid for two main reasons: first, for small loops time is lost in scanning empty space, and second, for some types of sample holders and bent loops a fixed size was often too small.

**Figure 2:**
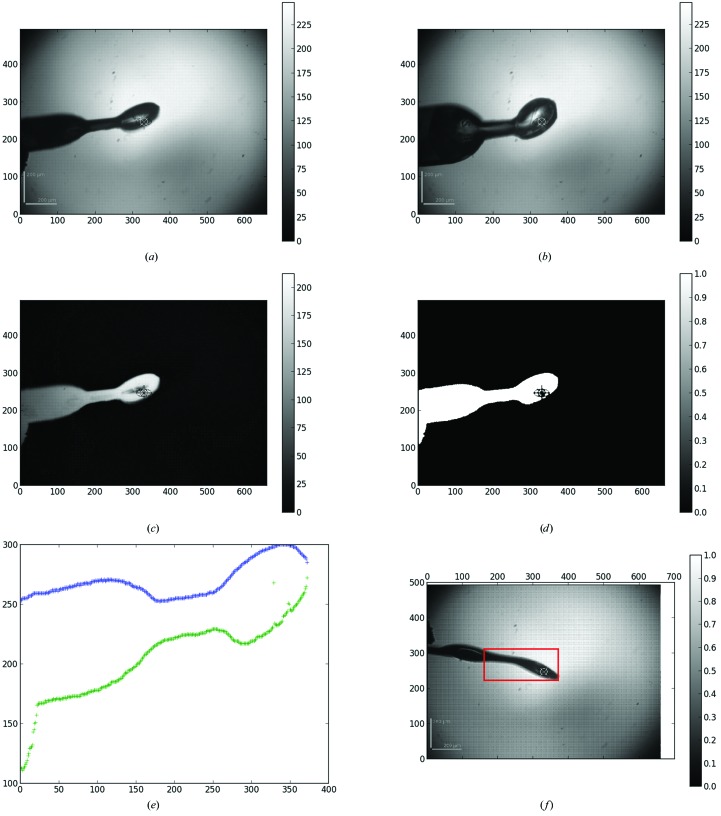
Image processing for the automesh algorithm. A series of images are taken 30° apart; examples are shown at 0° (*a*) and 90° (*b*). The background is then subtracted (*c*) and thresholding is used to detect the edges of the sample mount (*d*). The edges are then measured as a function of ω and pixels in order to determine the maximum and minimum ω orientations (*e*) and an area is selected for the mesh scan (*f*).

The automatic mesh algorithm can be used in two scenarios, where either the minimum or the maximum sample-mount size is determined as a function of rotation angle. The minimum grid size is used for sample holders containing a single crystal. The maximum grid size is used when locating more than one crystal in the sample holder in order to optimally detect all crystals. The input for the automatic determination of the mesh-scan area is a series of 12 images acquired from the online video microscope where the goniometer rotation axis is rotated by 30° between each image (Fig. 2 ▸ and Supplementary Movie). For each image, a background subtraction, Gaussian smoothing and the application of a threshold to find the contour (Figs. 2 ▸ *c* and 2 ▸ *d*) is performed. The contours of all 12 images are then analysed and the images corresponding to the minimum and maximum vertical sample support size are selected (Fig. 2 ▸ *e*). The corresponding rotation angle at which the mesh scan should be carried out and the size of the grid are returned as results. A snapshot of the area is also taken to ensure that the loop was covered in the mesh (Fig. 2 ▸ *f*).

**Figure 3:**
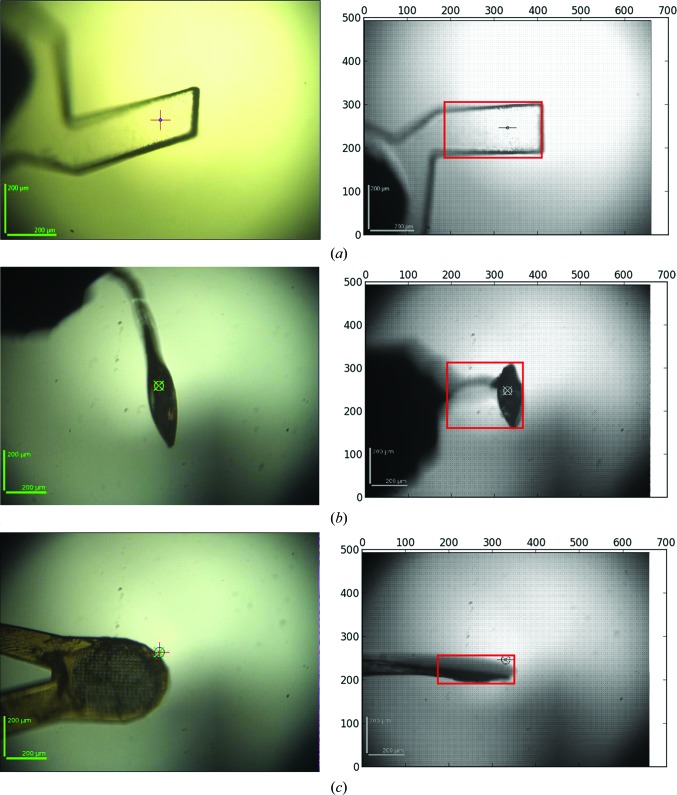
Automesh analysis of various sample holders. (*a*) A mount produced by laser-induced photoablation using the CrystalDirect robot (Cipriani *et al.*, 2012 ▸). (*b*) A bent loop. (*c*) A micromesh. The final area selected for the mesh scan is shown as a red box (right). In each case the entire mount is covered by the mesh scan and the vertical size is minimized in order to reduce the time taken for the scan.

The algorithm has allowed the automatic and optimal handling of many types of sample holder (Fig. 3 ▸). For small sample holders a minimum grid size is determined and for exotic sample holders, or bent loops, the grid is optimized to cover the whole sample holder while minimizing the mesh size. When the maximum size is selected, multiple crystals can be located and centred separately.

### 2.3. X-ray centring   

**Figure 4:**
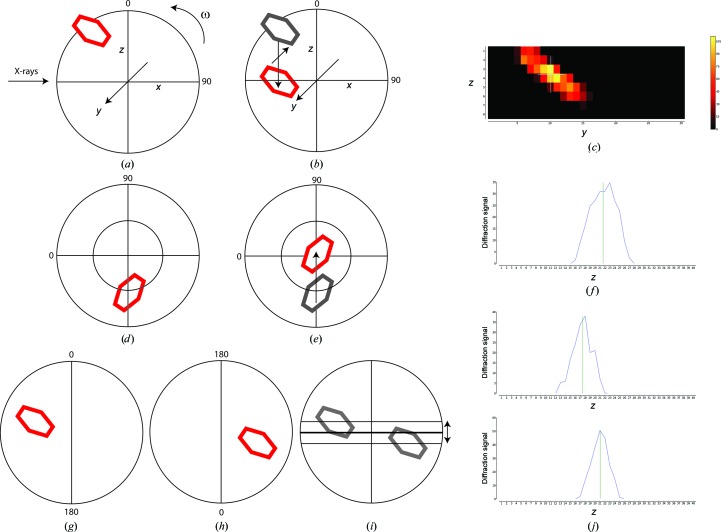
X-ray centring. (*a*) Once optical centring is completed, a crystal in a loop (red) will rotate about the goniometer centre of rotation (ω). A mesh scan determines the horizontal and vertical translations required to bring the crystal to the centre of rotation at this angle using the goniometer horizontal translation and the centring-table motors (*b*, *c*). Rotating the spindle by 90° (*d*) and performing a vertical scan determines the final movement of the centring-table motors required to place the optimum diffraction volume of the crystal at the centre of rotation of the spindle (*e*, *f*). In cases where the centre of rotation of the spindle is not placed at the beam position, it is determined by two vertical scans separated by 180° (*g*, *h*). The difference in position between the scans determines the vertical displacement of the goniometer required to place the centre of rotation of the spindle on the beam position (*i*, *j*). This example shows values of ω of 0 and 90° for convenience; arbitrary starting values of ω can be used.

The goal of X-ray centring is to locate and define, in three dimensions, the point within the crystal with the best diffraction signal and to place this point on the rotation axis. A two-dimensional mesh combined with a line scan, using only the centring-table and goniometer-translation motors, allows a point within the crystal to be centred over 360° while only sampling two orientations 90° apart (Fig. 4 ▸), making the routine extremely fast. The X-ray dose is minimized, with each position within the mesh receiving approximately 1 Gy, less than a single image in a complete data collection.

The two-dimensional mesh scan is performed over the area previously defined by analysis of the sample mount. The grid is divided into points, where the distances between points in the horizontal and vertical directions are defined by the beam size (either full beam or defined by the aperture preselected by the user; see §[Sec sec2.5]2.5). As the mesh is typically larger horizontally, and a delay is associated between measuring horizontal lines, the mesh is oversampled in the horizontal direction only. For each position, an image is acquired and processed using the program *Dozor*. The result of *Dozor* is a scalar value which estimates the diffraction signal of each image by combining an analysis of Bragg peaks with an intensity distribution as a function of resolution. Once all images have been processed by *Dozor*, the two-dimensional array containing the measured diffraction signal at each point is analysed (Fig. 4 ▸ *c*). The position of the best point is determined by first applying a threshold (50% of the maximum) to the signal and then performing a centre-of-mass calculation over any connected regions containing the strongest diffraction signal. The resulting position can be a fraction of the distance between the grid points. For crystals smaller than the vertical beam size it is necessary to perform a small vertical line scan to accurately position the sample as the two-dimensional mesh is not oversampled in the vertical direction. The decision to make this extra line scan is taken in cases where the best diffraction signal after the two-dimensional scan extends only one step in the vertical direction. If no signal is detected at this stage then the sample is unmounted.

After successful determination of the optimal position, the grid coordinates are translated into relative movements of the centring-table and goniometer-translation motors and the sample is moved to this position (Fig. 4 ▸ *b*). For a well calibrated goniometer this allows the sample to be centred over 360° by determining the position 90° away from the mesh scan with a single line scan (Figs. 4 ▸ *d*, 4 ▸ *e* and 4 ▸ *f*). However, in the case of MASSIF-1, the RoboDiff goniometer is both sample changer and goniometer. Therefore, the vertical position of the rotation axis must be determined and aligned to the X-ray beam, as it fluctuates between mounting cycles. This is determined using two scans of the goniometer with vertical translation 200 µm above and below the beam position performed 180° away from each other (Figs. 4 ▸ *g*– ▸ *j*). The centre of mass is then taken for each scan and the midpoint between these values defines the vertical offset required to ensure that a crystal is fully centred. This calculation uses segmentation to define the object of interest and the threshold is set at 25% of the maximum in order to define the crystal edges rather than the optimum diffraction points. Once determined, the axis is moved to the beam position and a corresponding vertical displacement of the sample is performed in order to maintain the optimal diffraction volume centred to the beam. The sample is then rotated 90° and a vertical line scan is performed on the centring table 400 µm above and below the beam position. After the sample has then been moved to the position with the strongest diffraction signal, it is centred on the rotation axis in the beam over the full rotation range (Fig. 4 ▸).

The X-ray centring procedure also provides measurements of crystal dimensions (height, width and depth) as a function of the rotation angle ω. This provides a simple model of the crystal volume used as input for strategy calculation (see §[Sec sec2.4]2.4). The automesh algorithm will generally orient a crystal such that its smallest vertical dimension is parallel to the rotation axis, allowing maximum and minimum dimensions to be defined. Occasionally, owing to sample mounting, crystals will not be optimally oriented, leading to small inaccuracies in the model of the crystal volume. However, having a rough estimate of volume relative to the beam size for correct dose estimations in strategy calculation is of enormous benefit and is superior to default values that generally bear no relationship to the actual crystal volume.

### 2.4. Strategy calculation and data collection   

At this stage, the workflow can be programmed to start a data collection with pre-defined parameters or to collect reference images in order to calculate an optimized strategy. The pre-defined parameters available are either a 180 or 360° rotation range with a 0.2° oscillation width and an exposure time based on a dose calculated from the measured flux (final dose of ∼31.3 MGy). After collection of data is completed, the workflow stops and the next sample is mounted.

If the workflow has not been programmed to start a fixed data-collection strategy, four reference images are collected 90° apart with predefined parameters of 100% transmission, 1° oscillation and an exposure time of 0.1 s, corresponding to a total dose of 10 Gy. The reference images are then analysed by the program *EDNA* (Incardona *et al.*, 2009 ▸), which uses a combination of *LABELIT* (Sauter *et al.*, 2004 ▸), *MOSLFM* (Leslie, 2006 ▸), *RADDOSE* (Paithankar *et al.*, 2009 ▸) and *BEST* (Bourenkov & Popov, 2010 ▸) to index the images and calculate an optimized data-collection strategy taking radiation damage into account. If reference images are not optimally collected (for example, using an incorrect exposure time, oscillation width and/or detector resolution), it can be difficult to calculate the optimal data-collection strategy. *EDNA*/*BEST* can suggest an optimized detector distance if required; therefore, the ability to automatically re-collect reference images with a different detector distance has been added to this process. *BEST* can predict from *B*-factor estimation the maximum resolution obtainable from a crystal. If the detector distance is below the value that *BEST* predicts, it will suggest a new distance at which to collect images. If this is the case, the characterization process is repeated with the new detector distance. In this way, optimal data are always collected. All data are subsequently processed automatically (Monaco *et al.*, 2013 ▸) using pipelines based on *XDS* (Kabsch, 2010 ▸) and, if a significant anomalous signal is detected, are also fed into SAD pipelines for structure determination. The results from all steps in the process are displayed in ISPyB. After collection of data is completed the workflow stops and the next sample can be mounted.

### 2.5. Pipelines and the diffraction plan   

The automation of processes necessarily involves standardization, such as the requirement for SPINE standard sample mounts. However, in order for an automatic data-collection service to be successful for a range of projects, considerable flexibility in data-collection parameters must be allowed. This has been implemented by combining different workflows with user input *via* the diffraction plan in the beamline database ISPyB. Here, information and specific data-collection requirements for each sample can be entered and pipelines for differing data-collection strategies can be selected (Table 1 ▸). Where no entry is given, a default value is used (Table 1 ▸). In this way, the characterization and data collection can be tailored to each individual sample.

Essential entries in the diffraction plan are the sample acronym (the protein being studied that has been approved by the ESRF safety group) and a unique sample name. Together, these items determine the nomenclature for the directories and diffraction images and, *via* the acronym, link to information about the crystal, such as space group and unit-cell parameters, if they have been entered. These are then used during the characterization step for strategy calculation and at the autoprocessing stage. The type of experiment can also be defined in the diffraction plan, with the user selecting default data collection (MXPressO), data collection using an *EDNA* strategy [MXPressE; a strategy optimized for 100% completeness with an 〈*I*/σ(*I*)〉 of 2 in the outer resolution shell taking radiation damage into account], SAD-optimized data collection [MXPressE SAD; a strategy optimized for high redundancy (360°) with the resolution set to where the *R*_merge_ between Bijvoet pairs is 5%, taking radiation damage into account] or characterization of diffraction properties without data collection (MXScore). The resolution that has already been observed is used as the basis for the detector distance for the mesh and line scans where characterization images are collected and is also used as the resolution for data collections where an *EDNA* strategy is not requested or indexing fails. A required resolution can also be entered; this value is used as a threshold below which data collection is not performed. Finally, the experiment can be further tailored to the samples by defining a required multiplicity or completeness or by specifying a certain radiation-sensitivity or beam size. The ability to add specific information at the level of the sample leads to a highly flexible system without compromising automation.

### 2.6. Error handling   

One of the most important aspects of an automated system is the correct handling of errors. Errors arising from robotic mounting of a sample are handled by low-level software which is designed to escape from the error and mount the next sample. Workflows are also paused when there is no beam (by monitoring the flux on a diode near the sample position) or the cryostream temperature rises above 120 K.

Each workflow executed by the BES has an overall error monitor. This ensures that workflows end cleanly in cases of software errors and automatically send an error report to a pre-configured mailing list. In the case of software errors, the current sample is unmounted and the process moves on to the next sample.

**Figure 5:**
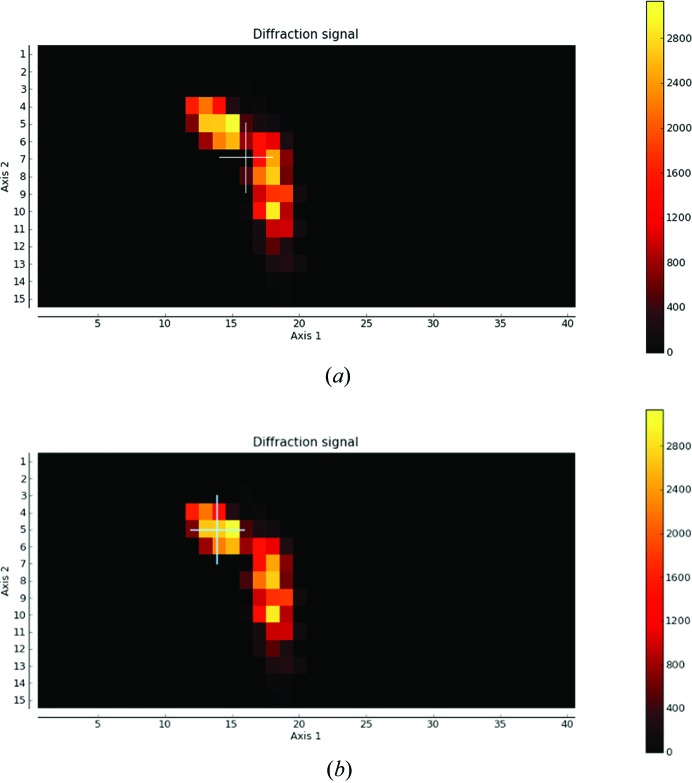
Error handling in the centre-of-mass calculation. The need to check for diffraction quality of the calculated centre of mass (white cross) is illustrated when the chosen position can be far from optimal (*a*). After verifying that the position chosen is within the threshold of the maximum, a new position is chosen (*b*).

More specific error handling exists for particular steps in the process. For the automatic determination of the area of mesh scans, if the algorithm fails (<1% for more than 1000 samples analysed) a default grid of 400 × 200 µm is returned. Errors can also occur during the selection of the optimum diffraction volume. As the centre of mass is often between grid points, this can sometimes result in a poor position being selected (Fig. 5 ▸ *a*). In order to avoid this scenario, the nearest image to the selected point is analysed and if the signal is less than 50% of the strongest position the process is repeated with the threshold increased by 5% until the selected position has the desired score (Fig. 5 ▸ *b*). In cases where *EDNA* characterization fails to produce a strategy, because, for example, the images cannot be indexed or integrated or *BEST* fails to calculate the intensity distribution correctly, a default strategy is used (180° rotation, 0.2° oscillation, final dose ∼31.3 MGy). Where a SAD strategy has been requested and fails, the default strategy is for 360° with the same final absorbed dose. In this way, even poorly diffracting samples have an associated data set, which can often yield useful information.

### 2.7. Chronometry   

**Figure 6:**
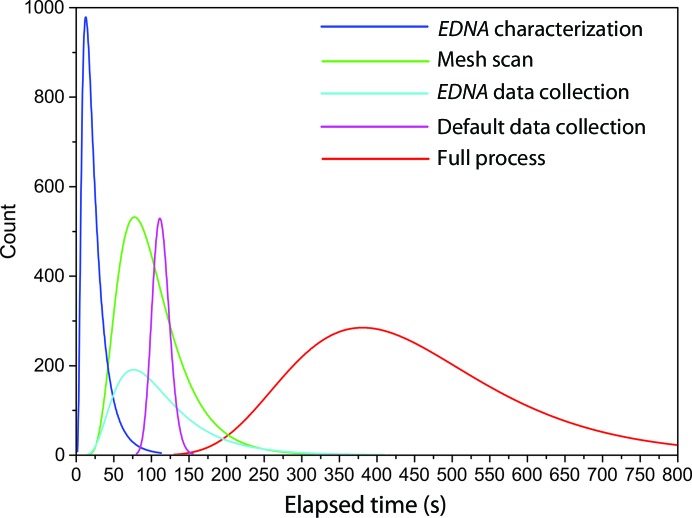
Log-normal distributions of durations for major steps in the automatic treatment of the first 1240 samples processed in 2015 on MASSIF-1.

The time taken to process each sample is important as it determines the number of samples that can be processed during scheduled beamtime. This varies depending on the size and shape of the mount and the size of the sample, as these will affect the time taken for the mesh scan and the eventual data-collection time. Fig. 6 ▸ and Table 2 ▸ show the distribution of durations for the major steps in the treatment of all samples processed in the first two months of 2015 (*N* = 1240) on MASSIF-1. The entire process takes an average of just over 7 min, with the most common time being just over 6 min. The longest steps in the process are the initial mesh scan (average of 101 s) and data collection (averages of 108 and 113 s for *EDNA* strategies and default collection, respectively). The time taken for default data collection has a very tight distribution, reflecting only small changes in the flux owing to differing ring currents. Data collections based on *EDNA* strategy calculations are far more varied and often take much less time (most frequently 72 s as opposed to 113 s), but there are also many with much longer data-collection times, showing that longer exposure times can be used when crystal size permits (Table 3 ▸). This will lead to higher signal-to-noise ratios and demonstrates the usefulness of data-collection strategy calculation.

## 3. Discussion   

The ability to collect data unattended should make the use of both available beam time and, it is hoped, the time of the users much more efficient. While completely automatic data collection can remove the more mundane tasks in data collection, it also presents new scientific opportunities. The degree of automation allows the experiments to be performed consistently with available details that are not normally used when scientists are present. An important example of this is the precise measurement of crystal dimensions. The X-ray centring procedure described is performed on all samples and not only consistently centres crystals to the optimal diffraction volume but also provides accurate information on crystal size. This information is then used by *RADDOSE* (Paithankar *et al.*, 2009 ▸) and *BEST* (Bourenkov & Popov, 2010 ▸) during *EDNA* characterization to calculate the absorbed dose used in a data collection. Properly defining crystal dimensions makes a considerable difference in the calculation of data-collection strategies (Table 3 ▸), leading to increased resolution and signal-to-noise ratios for crystals larger than the X-ray beam and reduced radiation damage for crystals smaller than the X-ray beam. The accurate measurement of crystal dimensions coupled to the real-time measurement of the flux of the X-ray beam leads to the best possible data being collected for a given sample when *EDNA* characterization is used.

**Figure 7:**
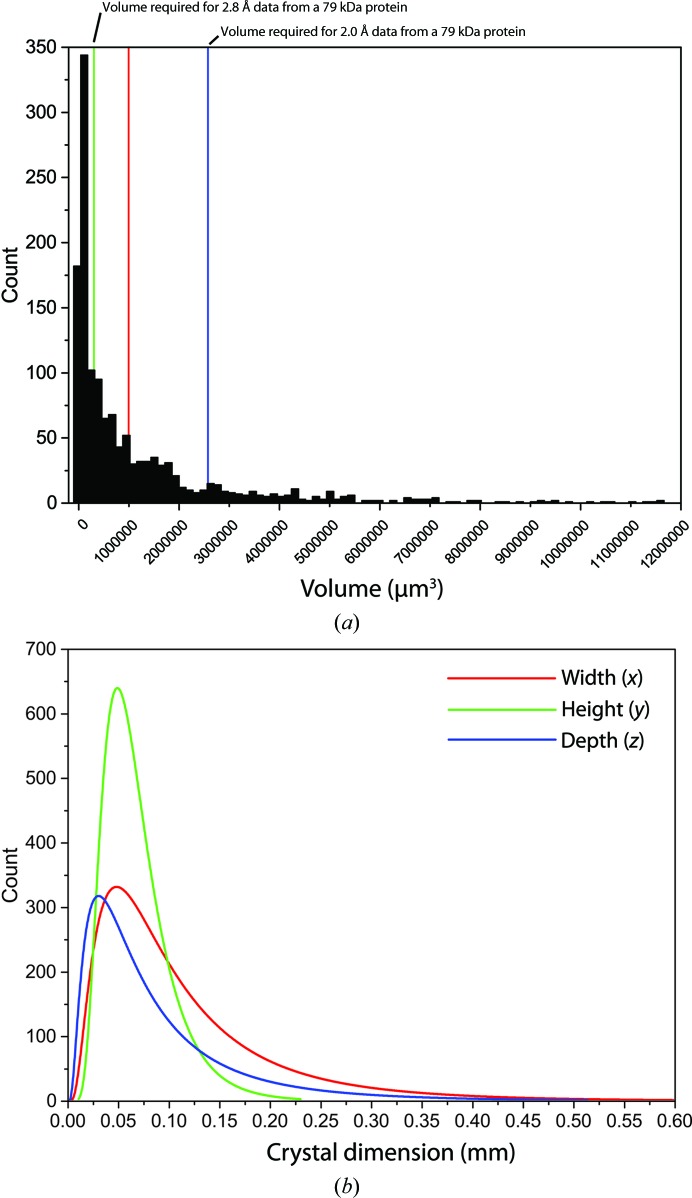
Distributions of crystal volumes and dimensions observed for the first 1240 samples processed in 2015 on MASSIF-1. (*a*) Histogram of crystal volumes; 30% of samples fall below the average volume of ∼10^6^ µm^3^ (red line). Theoretical crystal volumes (Holton & Frankel, 2010 ▸) required for data at 2.0 Å (blue line) or 2.8 Å (green line) resolution for a protein of 79 kDa on MASSIF-1 are shown for reference. Volume data are shown only to 1.2 × 10^7^ µm^3^ for clarity. (*b*) Log-normal distributions of crystal dimensions. The dimensions are *x*, the measured crystal length parallel to the spindle axis, *y*, the length orthogonal to the spindle axis, and *z, * the length orthogonal to the spindle axis 90° away in ω.

The consistent measurement of all parameters related to not only each experiment but also to whole projects will allow information to be fed back into future experiments. For example, the analysis of the crystal dimensions the first 1240 crystals processed on MASSIF-1 in 2015 is informative (Table 4 ▸ and Fig. 7 ▸). The average values for crystal volume and individual dimensions are as expected, being roughly 100 µm. However, the histogram of measured volumes is striking in that most crystals are much smaller than this average (Fig. 7 ▸ *a*), and this is also reflected in the distributions of individual dimensions, with peaks below 50 µm in all three dimensions (Fig. 7 ▸ *b* and Table 4 ▸). Over 44% of the crystals processed on MASSIF-1 are below 250 000 µm in volume, equivalent to a cube with sides of 63 µm. The most commonly observed volume is 20 209 µm^3^ (edges of ∼27 µm), which is within two orders of magnitude of the theoretical limit of a 8.3 µm diameter required to obtain 2 Å resolution data from a ‘realistic’ lysozyme crystal (Holton & Frankel, 2010 ▸). In fact, half of the crystals so far processed on MASSIF-1 in 2015 are smaller in volume than the size theoretically required to obtain 2.8 Å resolution data from a protein crystal with an entity of 79 kDa (PDB average) in the asymmetric unit (304 980 µm^3^; Holton & Frankel, 2010 ▸), showing that the beamline is working at the leading edge of scientific projects and is not just for well diffracting samples; it is also heavily used for screening. This analysis has led to the selection of a default beam diameter of 50 µm, as this most accurately reflects the size of most crystals observed so far on the beamline. The distributions also reflect the tendency of crystals to lie along the spindle axis owing to the way they are mounted, leading to the height of the crystals having the smallest range (Fig. 7 ▸ *b*). Analysis of this type should also prove useful for specific projects. Reports can be generated showing crystal size distribution and variation in diffraction quality (Bowler & Bowler, 2014 ▸) for each protein. This information can then be fed back into projects, informing, for example, on whether larger or smaller crystals are better and the optimal selection of beam size, depending on variation in diffraction.

Fully automatic characterization and data collection from large numbers of crystals is a new tool for structural biologists. The process is used heavily at opposing ends of the project spectrum. In the initial stages of projects, preliminary hits from high-throughput crystallization or large numbers of poor-quality crystals need to be screened, a time-intensive stage that results in little data collection but requires rapid feedback. The opposing use is the collection of hundreds of data sets from well established systems, as is the case in small-molecule fragment-screening projects. Again, this process is labour-intensive but requires more effort in the interpretation of results and again requires rapid feedback. The process described here facilitates both stages, allowing effort to be redirected from the beamline to the laboratory. In combination with recent developments in the automation of crystal mounting (Cipriani *et al.*, 2012 ▸), it is hoped that project lifecycles can be reduced, leading to an increase in the quantity and the quality of structural results available.

## Supplementary Material

Supplementary movie displaying the process to determine the mesh scan area.. DOI: 10.1107/S1399004715011918/wa5095sup1.wmv

## Figures and Tables

**Table 1:** Diffraction-plan entries The diffraction plan is filled for each sample in ISPyB and is used by the system to tailor the experiment to the sample. In the absence of an entry, sensible default values are used.

Diffraction-plan entry	Definition	Default value
Protein acronym	Defines the protein that is registed with the ESRF safety group	Required field
Sample name	User-defined unique identifier	Required field
Pin barcode	Barcode identifier	None
Experiment type	Define MXPressE/O/SAD/Score	MXPressE
Space group	If present used for strategy calculation and autoprocessing	None
Pre-observed resolution	Resolution that the detector will be set to for mesh scans, characterization images and default data collection	2.0
Required resolution	Threshold resolution; samples below the cutoff will not be collected	None
Radiation-sensitivity	*BEST* input in the case of highly radiation-sensitive crystals (0.52.0 for high to low sensitivity)	1
Required completeness		99%
Required multiplicity		4
No. of positions	For multiple crystals	1
Preferred beam size	Select appropriate beam size for crystals	50m

**Table 2:** Duration of steps in the automatic treatment of samples on MASSIF-1 The values are for the first 1240 samples processed in 2015.

Step	Mesh scan (s)	*EDNA* characterization (s)	*EDNA* data collection (s)	Default data collection (s)	Workflow total time (s)
Mean	101	25	108	113	440
Mode	62	9	72	113	395
Median	85	22	95	112	444
Maximum	299	138	316	138	993
Minimum	35	5	14	109	85
*N*	1240	1147	552	551	1240

**Table 3:** The use of crystal dimensions in strategy calculation Differences in the calculated strategy to obtain native data with an *I*/(*I*) of 3.0 in the outer resolution shell based on the measured crystal size or the default crystal size in *RADDOSE* and *BEST*. The strategies are taken from the largest and smallest crystals processed with a strategy on MASSIF-1 in 2015.

	Crystal larger than beam		Crystal smaller than beam	
	Default crystal size	Measured crystal size	Default crystal size	Measured crystal size
Crystal dimensions (m)	100 100 100	603 238 397	100 100 100	74 37 13
Beam diameter (m)	50	50	50	50
Space group	*C*2_1_	*C*2_1_	*P*222	*P*222
Unit-cell parameters				
*a* ()	129.00	129.00	86.37	86.37
*b* ()	208.38	208.38	90.66	90.66
*c* ()	117.50	117.50	113.25	113.25
()	109.3	109.3	90	90
= ()	90	90	90	90
Flux (photonss^1^)	1.2 10^12^	1.2 10^12^	1.2 10^12^	1.2 10^12^
Transmission (%)	100	100	100	54.6
Dose (MGy)	13.23	36.38	13.54	4.93
Total exposure time (s)	178.9	507.1	91.5	60.4
Oscillation range ()	108252	108252	170275	9160
Detector resolution ()	2.16	2.05	2.86	3.06

**Table 4:** Crystal dimensions observed on MASSIF-1 The values are for the first 1240 samples processed in 2015.

Crystal dimension	Mean	Mode	Maximum	Minimum
Width *x* (m)	119	37	828	28
Height *y* (m)	69	29	374	18
Depth *z* (m)	90	26	513	13
Volume (m^3^)	1629310	20209	56735667	8297
Cube root of volume (m)	118	27	384	20
Actual minimum and maximum dimensions (m)			*x* = 603, *y* = 238, *z* = 397	*x* = 36, *y* = 18, *z* = 13

## References

[bb1] Aishima, J., Owen, R. L., Axford, D., Shepherd, E., Winter, G., Levik, K., Gibbons, P., Ashton, A. & Evans, G. (2010). *Acta Cryst.* D**66**, 1032–1035. 10.1107/S0907444910028192 PMC6691516 20823554

[bb2] Arzt, S. *et al.* (2005). *Prog. Biophys. Mol. Biol.* **89**, 124–152. 10.1016/j.pbiomolbio.2004.09.003 15910915

[bb3] Bedem, H. van den, Wolf, G., Xu, Q. & Deacon, A. M. (2011). *Acta Cryst.* D**67**, 368–375. 10.1107/S0907444910039934 PMC3069752 21460455

[bb4] Ben-Shem, A., Frolow, F. & Nelson, N. (2003). *Nature (London)*, **426**, 630–635. 10.1038/nature02200 14668855

[bb5] Ben-Shem, A., Jenner, L., Yusupova, G. & Yusupov, M. (2010). *Science*, **330**, 1203–1209. 10.1126/science.1194294 21109664

[bb6] Berman, H. M., Coimbatore Narayanan, B., Costanzo, L. D., Dutta, S., Ghosh, S., Hudson, B. P., Lawson, C. L., Peisach, E., Prlić, A., Rose, P. W., Shao, C., Yang, H., Young, J. & Zardecki, C. (2013). *FEBS Lett* **587**, 1036–1045. 10.1016/j.febslet.2012.12.029 PMC4068610 23337870

[bb7] Beteva, A. *et al.* (2006). *Acta Cryst.* D**62**, 1162–1169. 10.1107/S0907444906032859 17001093

[bb8] Bourenkov, G. P. & Popov, A. N. (2010). *Acta Cryst.* D**66**, 409–419. 10.1107/S0907444909054961 PMC2852305 20382994

[bb9] Bowler, M. G. & Bowler, M. W. (2014). *Acta Cryst.* F**70**, 127–132. 10.1107/S2053230X13032007 PMC3943112 24419635

[bb10] Bowler, M. W., Guijarro, M., Petitdemange, S., Baker, I., Svensson, O., Burghammer, M., Mueller-Dieckmann, C., Gordon, E. J., Flot, D., McSweeney, S. M. & Leonard, G. A. (2010). *Acta Cryst.* D**66**, 855–864. 10.1107/S0907444910019591 20693684

[bb11] Brockhauser, S., Svensson, O., Bowler, M. W., Nanao, M., Gordon, E., Leal, R. M. F., Popov, A., Gerring, M., McCarthy, A. A. & Gotz, A. (2012). *Acta Cryst.* D**68**, 975–984. 10.1107/S090744491201863X PMC3413211 22868763

[bb12] Cherezov, V., Hanson, M. A., Griffith, M. T., Hilgart, M. C., Sanishvili, R., Nagarajan, V., Stepanov, S., Fischetti, R. F., Kuhn, P. & Stevens, R. C. (2009). *J. R. Soc. Interface*, **6**, S587–S597. 10.1098/rsif.2009.0142.focus PMC2843980 19535414

[bb13] Cipriani, F. *et al.* (2006). *Acta Cryst.* D**62**, 1251–1259. 10.1107/S0907444906030587 17001102

[bb14] Cipriani, F., Röwer, M., Landret, C., Zander, U., Felisaz, F. & Márquez, J. A. (2012). *Acta Cryst.* D**68**, 1393–1399. 10.1107/S0907444912031459 22993093

[bb15] Cohen, A. E., Ellis, P. J., Miller, M. D., Deacon, A. M. & Phizackerley, R. P. (2002). *J. Appl. Cryst.* **35**, 720–726. 10.1107/s0021889802016709 PMC4041710 24899734

[bb16] Dauter, Z. (1999). *Acta Cryst.* D**55**, 1703–1717. 10.1107/s0907444999008367 10531520

[bb17] Delagenière, S. *et al.* (2011). *Bioinformatics*, **27**, 3186–3192. 10.1093/bioinformatics/btr535 21949273

[bb18] Deller, M. C. & Rupp, B. (2014). *Acta Cryst.* F**70**, 133–155. 10.1107/S2053230X14000387 PMC3936438 24637746

[bb19] Elsliger, M.-A., Deacon, A. M., Godzik, A., Lesley, S. A., Wooley, J., Wüthrich, K. & Wilson, I. A. (2010). *Acta Cryst.* F**66**, 1137–1142. 10.1107/S1744309110038212 PMC2954196 20944202

[bb20] Ferrer, J.-L., Larive, N. A., Bowler, M. W. & Nurizzo, D. (2013). *Exp. Opin. Drug. Discov.* **8**, 835–847. 10.1517/17460441.2013.793666 23656378

[bb21] Gabadinho, J. *et al.* (2010). *J. Synchrotron Rad.* **17**, 700–707. 10.1107/S0909049510020005 PMC3025540 20724792

[bb22] Heikkila, T., Surade, S., Silvestre, H., Dias, M. B., Ciulli, A., Bromfield, K., Scott, D., Howard, N., Wen, S., Wei, A., Osborne, D., Abell, C. & Blundell, T. (2009). *From Molecules to Medicines: Structure of Biological Macromolecules and Its Relevance in Combating New Diseases and Bioterrorism*, edited by J. Sussman & P. Spadon, pp. 21–36. Dordrecht: Springer.

[bb23] Heinemann, U., Büssow, K., Mueller, U. & Umbach, P. (2003). *Acc. Chem. Res.* **36**, 157–163. 10.1021/ar010129t 12641472

[bb24] Hilgart, M. C., Sanishvili, R., Ogata, C. M., Becker, M., Venugopalan, N., Stepanov, S., Makarov, O., Smith, J. L. & Fischetti, R. F. (2011). *J. Synchrotron Rad.* **18**, 717–722. 10.1107/S0909049511029918 PMC3161817 21862850

[bb25] Holton, J. & Alber, T. (2004). *Proc. Natl Acad. Sci. USA*, **101**, 1537–1542. 10.1073/pnas.0306241101 PMC341770 14752198

[bb26] Holton, J. M. & Frankel, K. A. (2010). *Acta Cryst.* D**66**, 393–408. 10.1107/S0907444910007262 PMC2852304 20382993

[bb27] Incardona, M.-F., Bourenkov, G. P., Levik, K., Pieritz, R. A., Popov, A. N. & Svensson, O. (2009). *J. Synchrotron Rad.* **16**, 872–879. 10.1107/S0909049509036681 19844027

[bb28] Jacquamet, L., Joly, J., Bertoni, A., Charrault, P., Pirocchi, M., Vernede, X., Bouis, F., Borel, F., Périn, J.-P., Denis, T., Rechatin, J.-L. & Ferrer, J.-L. (2009). *J. Synchrotron Rad.* **16**, 14–21. 10.1107/S0909049508031105 19096169

[bb29] Kabsch, W. (2010). *Acta Cryst.* D**66**, 125–132. 10.1107/S0907444909047337 PMC2815665 20124692

[bb30] Leslie, A. G. W. (2006). *Acta Cryst.* D**62**, 48–57. 10.1107/S0907444905039107 16369093

[bb31] Malbet-Monaco, S., Leonard, G. A., Mitchell, E. P. & Gordon, E. J. (2013). *Acta Cryst.* D**69**, 1289–1296. 10.1107/S0907444913001108 PMC3689532 23793155

[bb32] Monaco, S., Gordon, E., Bowler, M. W., Delagenière, S., Guijarro, M., Spruce, D., Svensson, O., McSweeney, S. M., McCarthy, A. A., Leonard, G. & Nanao, M. H. (2013). *J. Appl. Cryst.* **46**, 804–810. 10.1107/S0021889813006195 PMC3654316 23682196

[bb33] Ohana, J., Jacquamet, L., Joly, J., Bertoni, A., Taunier, P., Michel, L., Charrault, P., Pirocchi, M., Carpentier, P., Borel, F., Kahn, R. & Ferrer, J.-L. (2004). *J. Appl. Cryst.* **37**, 72–77.

[bb34] Okazaki, N., Hasegawa, K., Ueno, G., Murakami, H., Kumasaka, T. & Yamamoto, M. (2008). *J. Synchrotron Rad.* **15**, 288–291. 10.1107/S0909049507064679 PMC2394786 18421161

[bb35] Paithankar, K. S., Owen, R. L. & Garman, E. F. (2009). *J. Synchrotron Rad.* **16**, 152–162. 10.1107/S0909049508040430 19240327

[bb36] Panjikar, S., Parthasarathy, V., Lamzin, V. S., Weiss, M. S. & Tucker, P. A. (2005). *Acta Cryst.* D**61**, 449–457. 10.1107/S0907444905001307 15805600

[bb37] Perrakis, A., Morris, R. & Lamzin, V. S. (1999). *Nature Struct. Biol.* **6**, 458–463. 10.1038/8263 10331874

[bb38] Pohl, E., Ristau, U., Gehrmann, T., Jahn, D., Robrahn, B., Malthan, D., Dobler, H. & Hermes, C. (2004). *J. Synchrotron Rad.* **11**, 372–377. 10.1107/S090904950401516X 15310952

[bb39] Robinson, H., Soares, A. S., Becker, M., Sweet, R. & Héroux, A. (2006). *Acta Cryst.* D**62**, 1336–1339. 10.1107/S0907444906026321 17057336

[bb40] Sauter, N. K., Grosse-Kunstleve, R. W. & Adams, P. D. (2004). *J. Appl. Cryst.* **37**, 399–409. 10.1107/S0021889804005874 PMC2808709 20090869

[bb41] Selmer, M., Dunham, C. M., Murphy, F. V., Weixlbaumer, A., Petry, S., Kelley, A. C., Weir, J. R. & Ramakrishnan, V. (2006). *Science*, **313**, 1935–1942. 10.1126/science.1131127 16959973

[bb42] Soltis, S. M. *et al.* (2008). *Acta Cryst.* D**64**, 1210–1221. 10.1107/S0907444908030564 PMC2631117 19018097

[bb43] Tsai, Y., McPhillips, S. E., González, A., McPhillips, T. M., Zinn, D., Cohen, A. E., Feese, M. D., Bushnell, D., Tiefenbrunn, T., Stout, C. D., Ludaescher, B., Hedman, B., Hodgson, K. O. & Soltis, S. M. (2013). *Acta Cryst.* D**69**, 796–803. 10.1107/S0907444913001984 PMC3640469 23633588

[bb44] Ueno, G., Hirose, R., Ida, K., Kumasaka, T. & Yamamoto, M. (2004). *J. Appl. Cryst.* **37**, 867–873.

[bb45] Warne, T., Serrano-Vega, M. J., Baker, J. G., Moukhametzianov, R., Edwards, P. C., Henderson, R., Leslie, A. G. W., Tate, C. G. & Schertler, G. F. (2008). *Nature (London)*, **454**, 486–491. 10.1038/nature07101 PMC2923055 18594507

[bb46] Wasserman, S. R., Koss, J. W., Sojitra, S. T., Morisco, L. L. & Burley, S. K. (2012). *Trends Pharmacol. Sci.* **33**, 261–267. 10.1016/j.tips.2012.03.009 22521107

[bb47] Zouni, A., Witt, H. T., Kern, J., Fromme, P., Krauss, N., Saenger, W. & Orth, P. (2001). *Nature (London)*, **409**, 739–743. 10.1038/35055589 11217865

